# Assessment of Knowledge, Awareness, and Perceptions of Cancer Among Accredited Social Health Activists (ASHAs): A Multistate Qualitative Study From India

**DOI:** 10.7759/cureus.105681

**Published:** 2026-03-23

**Authors:** Yashraj A Birari, Ansu P Koshy, Mohit Kumar, Kulsum Jaffry, Sherin Juditta, Paul P Francis, Deepshikha Kamra, Pamela A Jeyaraj, Clarence J Samuel

**Affiliations:** 1 Department of Community Medicine, Christian Medical College and Hospital, Ludhiana, IND; 2 Department of Public Health, Garo Hills Adventist Mission Hospital, Jengjal, Jengjal, IND; 3 Department of Radiation Oncology, Christian Medical College and Hospital, Ludhiana, IND

**Keywords:** ashas, breast cancer, cancer awareness, cervical cancer, oral cancer, primary healthcare, qualitative result

## Abstract

Background

This qualitative study aims to assess the knowledge, awareness, attitudes, beliefs, and perceived barriers related to cancer prevention and screening among Accredited Social Health Activist (ASHA) workers across three Indian states (Punjab, Uttar Pradesh, and Meghalaya).

Methods

Focus group discussions (FGDs) were conducted with ASHA workers using a semi-structured guide covering awareness of cancer symptoms, screening practices, risk perception, and cultural beliefs. The study included a total of 90 ASHA workers, of whom 50 were from Ludhiana, 25 from Uttar Pradesh, and 15 from Meghalaya. Data were thematically analyzed using ATLAS.ti software (Scientific Software Development GmbH, Berlin, Germany). Themes were coded across categories, including general awareness, screening behavior, personal experience, trusted information sources, and community recommendations.

Results

ASHAs demonstrated partial but varied knowledge of cancer symptoms, with higher awareness of oral cancer compared to breast and cervical cancers. Key barriers to screening included fear of infection, stigma, lack of facility availability, and sociocultural myths. Despite recognizing the benefits of early detection, most ASHAs had not undergone screening themselves. Personal experience with cancer cases led to greater engagement. ASHAs suggested community-based interventions such as free screening camps, collaboration with religious centers, and enhanced government support.

Conclusion

While ASHAs have the potential to serve as change agents for cancer control at the grassroots level, their knowledge and practices are constrained by systemic, educational, and cultural barriers. Strengthening their capacity through standardized oncology training, improving primary healthcare infrastructure, and integrating noncommunicable disease (NCD) services into Health and Wellness Centers are key steps toward improving cancer outcomes in India.

## Introduction

Cancer is a leading cause of death worldwide and remains a major challenge to improving life expectancy and quality of life. Globally, cancer is the second leading cause of mortality, causing one death in every six [1]. Breast and cervical cancers account for almost one million deaths each year, disproportionately high in low- and middle-income countries (LMICs). In India, breast cancer is the leading cancer among women, accounting for 28%, followed by cervical cancer (16%). Together, they account for about 250,000 new cases and close to 100,000 deaths each year, highlighting their significant public health impact [2].

The Global Action Plan of the World Health Organization (WHO) for the Prevention and Control of Non-communicable Diseases (2013-2020) identifies screening, early detection, and early treatment of cancers - breast, oral, colorectal, and cervical - as a cost-effective intervention for LMICs [3]. By 2030, it is projected that there will be approximately 26 million new cancer cases and 17 million cancer deaths per year. The greatest burden of cancer-related deaths will be in low-income countries, which is up to five times higher than in high-income nations [4]. These projections underline the urgent need for strengthening preventive and early diagnostic strategies.

One of the major contributors to delayed diagnosis and poor outcomes is the absence or inadequate implementation of organized nationwide screening programs. In India, disparities exist across regions, with certain states reporting particularly high disease burden. For example, cervical cancer remains a significant concern in the northeastern states; in Meghalaya, it is the second most common cancer among women, accounting for 11.2% of female cancer cases [5]. Research suggests that a poor prognosis and delayed diagnosis are mostly caused by a lack of awareness of cancer symptoms, risk factors, and screening techniques. Because of stigma, fear, misunderstandings, and a low perceived risk of illness, screening services are frequently underutilized, even in environments where they are offered. Attitude barriers have a major impact on health-seeking behavior and contribute to late-stage presentation, which lowers the efficacy of cancer control programs, especially among women in poor and resource-limited regions.

With increasing rates of incidence and mortality, cancers such as breast, cervical, and oral cancer have emerged as a serious health concern in India. Despite the advancement in medical technology, early detection and timely intervention are still challenging due to sociocultural beliefs, educational disparities, and inadequate healthcare access. Stigma and misconceptions also result in diagnostic delay. Understanding the level of knowledge and attitudes toward common cancers is essential for designing effective health education programs and improving participation in screening initiatives. However, there is still a significant lack of region-specific information regarding screening procedures, risk factors, and cancer symptom knowledge, especially among underprivileged groups. The capacity of public health systems to create focused, culturally relevant treatments is hampered by the absence of localized evidence. Thus, the purpose of this study was to evaluate attitudes and knowledge regarding prevalent malignancies and to find any gaps that might guide focused, evidence-based approaches to enhancing cancer prevention, early detection, and screening uptake.

## Materials and methods

This study employed a qualitative exploratory design using focus group discussions (FGDs) to examine the knowledge, perceptions, and attitudes of Accredited Social Health Activists (ASHAs) regarding breast, cervical, and oral cancers. The study was conducted across three socio-culturally diverse states in India - Punjab, Uttar Pradesh, and Meghalaya - to capture geographical variations in access to healthcare, cultural beliefs, and awareness related to cancer. A total of 90 ASHA workers participated in the study, including 50 from Ludhiana (Punjab), 25 from Shahjahanpur (Uttar Pradesh), and 15 from Garo Hills (Meghalaya). A purposive sampling approach was used to recruit active frontline ASHA workers who were available during the data collection period and could provide diverse perspectives on cancer awareness and community perceptions. A total of nine FGDs were conducted, with three FGDs in each state. Each FGD consisted of 8-10 ASHA workers. Only active frontline health workers who were present and willing to participate at the time of data collection were included in the study.

FGDs used a pre-formulated guide, exploring general knowledge on cancer, interpretation of symptoms and risk factors, personal and community-level experience, screening practice, attitude towards treatment, and typical cultural beliefs. The research project started on 15 February 2024, and the expected end date is March 2027. An interview guide was developed after an extensive review of relevant literature and consultation with subject experts to ensure content relevance and alignment with study objectives. The draft guide was pilot tested on a small group of participants similar to the study population to assess clarity, flow, and understanding of questions. Based on feedback from the pilot testing, necessary modifications were made to improve wording and sequencing. The revised interview guide was then validated through expert review to establish content validity, ensuring that the questions adequately captured the intended domains of inquiry. The FGD was conducted in Punjabi, Hindi, English, and Garo, depending on the language spoken at the site and the choice of respondents. All FGDS were audio-recorded after obtaining informed consent. These were supplemented by the field notes, which captured non-verbal data and information that may assist in the interpretation of local terminology (see Appendices).

After the FGDs, all audio recordings were transcribed and translated into English to ensure consistency in analysis. The transcripts were carefully reviewed several times to familiarize the researchers with the data. Thematic coding was carried out using ATLAS.ti software (version 7; Scientific Software Development GmbH, Berlin, Germany) to facilitate systematic data organization and analysis. Initial open coding was performed to identify meaningful segments of text related to ASHA workers’ knowledge, perceptions, and attitudes toward cancer. Codes were generated inductively from the data and subsequently grouped into broader thematic categories. To enhance analytical rigor, the coding process was reviewed by members of the research team, and differences in interpretation were discussed and resolved through consensus. The codes were then organized into broader thematic families such as cancer knowledge, sources of information, screening practices, barriers to screening, personal experiences, attitudes and beliefs, trusted sources of information, and suggestions for improving cancer awareness. Codes in these categories included symptoms (e.g., lumps, watery discharge), varying degrees of knowledge of cancer, hospital consultations or courses as sources of information, perceptions regarding causes of cancer (e.g., tobacco consumption, hygiene, lifestyle), and prevention and early detection attitudes. Fear of infection, myths, and lack of awareness were the most frequently mentioned barriers, and suggestions made included organizing free treatment camps, health campaigns at the community level, and making screening easier to access.

The analysis was descriptive and progressive, with ideas always developing and being improved. Coding and interpretation proceeded simultaneously to ensure that the socio-cultural context was not lost and remained central to findings. This study was approved by the Institutional Ethics Committee - Biomedical Research of Christian Medical College and Hospital, Ludhiana. Confidentiality and privacy were maintained during the process. Data were anonymized prior to analysis.

## Results

The results indicated a heterogeneous view of cancer among ASHA workers, defined by variation in awareness, cultural beliefs, personal experience, and barriers to access. Analysis of FGD provided several salient themes, such as general awareness, screening and prevention, personal experience, attitudes and beliefs, information sources, and community-level suggestions for improving cancer education and screening uptake.

Table 1 summarizes the demographic and professional characteristics of the study participants, including age, educational level, marital status, occupation, and monthly family income. These characteristics provide essential context for interpreting participants' perspectives and potential factors influencing the responses.

**Table 1:** Sociodemographic Characteristics of ASHAs Data are presented as a number (percentage). Age is presented as mean ± standard deviation (SD). ASHA: Accredited Social Health Activist; INR: Indian Rupees

Variable	Category	Value
Age	43±9.53	-
Gender	Female	90 (100)
Marital Status	Married	85 (94.4)
	Unmarried	3 (3.3)
	Widowed	2 (2.2)
	Total	90 (100)
Occupation Status	Employed	84 (93.3)
	Unemployed	6 (6.7)
	Total	90 (100)
Education	Illiterate	1 (1.1)
	Primary School	2 (2.2)
	Middle School	25 (27.8)
	High School	33 (36.7)
	Intermediate/Diploma	8 (8.9)
	Graduate	21 (23.3)
	Total	90 (100)
Monthly Family Income	<10,000 INR	38 (42.2)
	INR 10,001-20,000	25 (27.8)
	INR 20,001-50,000	13 (14.4)
	>50,000 INR	14 (15.6)
	Total	90 (100)

The study included a total of 90 ASHA workers, of whom 50 were from Ludhiana, 25 from Uttar Pradesh, and 15 from Meghalaya.

Table 2 summarizes the major themes and subthemes emerging from the qualitative analysis of ASHAs’ perceptions regarding breast, cervical, and oral cancers, along with key insights and illustrative participant quotes. It captures their levels of awareness, beliefs, screening practices, personal experiences, perceived barriers, and suggestions for improving cancer awareness, prevention, and early detection.

**Table 2:** Thematic Summary of ASHA Workers’ Knowledge and Perceptions of Cancer ASHAs: Accredited Social Health Activists; CHCs: community health centres; CHOs: community health officers

Theme	Subtheme	Key Insights	Representative Quote (Italicized)
General Awareness	Symptom Knowledge	Most ASHAs recognized key signs of breast (lump, discharge), cervical (irregular bleeding), and oral (ulcers, tobacco use) cancers.	“When there is a lump in the breast or watery discharge coming out.”
	Knowledge Level	Mixed levels: Some confident, others admitted limited knowledge.	“We have little information about these cancers, but whatever we have, we make people aware.”
	Information Sources	Hospitals, CHCs, training sessions, and CHOs were most cited.	“Doctor told us in the training session.”
Screening and Prevention	Barriers to Screening	Fear of infection and sociocultural myths were major barriers.	“I also had a case 8 years ago, the patient fell under the influence of a baba. The patient did not go to the doctor and stayed at home”
	Screening Practices	Majority of ASHAs had not undergone screening themselves.	“No, ma’am.” - Common response across all regions
Personal Experience	Cancer Cases Known	Mixed exposure: some had direct or indirect experiences, others had none.	“There is a patient in my family… but now the patient has died.”
Attitudes and Beliefs	Cause of Cancer	Common beliefs: tobacco use, poor hygiene, lifestyle. Some misconceptions present.	“Yes ma’am, lifestyle habits are the cause of these cancers.”
	Prevention Beliefs	Some believed cancer is preventable; others doubtful or skeptical.	“Yes, it can be prevented.”/“No, ma’am.”
	Early Detection Benefits	Strong belief in the role of early detection in improving survival.	“If diagnosis is done on time, then with treatment, we will get prolonged life.”
Trusted Information	Media and Outreach	Posters, mobile ads, educated individuals, workshops mentioned.	“Many advertisements are shown on mobiles… people gather information through posters.”
Suggestions	Community Recommendations	Need for free screening camps, more outreach, government support.	“There should be screening camps and free treatments.”

Under the rubric of general awareness, most of the ASHA workers had a poor understanding of the signs of breast, cervical, and oral cancer. Breast cancer knowledge was commonly associated with having a lump or watery discharge. Cervical cancer was commonly equated with abnormal menstruation and irregular bleeding. Oral cancer was equated with the consumption of tobacco, mouth ulcers, mouth wounds, and poor oral hygiene. One of the ASHA workers in Pakhowal stated, "When there is a lump in the breast or watery discharge coming out," which indicated that symptoms were recognized. Others mentioned other symptoms such as irregular menstrual periods or abnormal vaginal bleeding. Some of the ASHAs, however, acknowledged that they did not know a lot. "We don't know much about these cancers, but what we do, we inform people," commented a 38-year-old ASHA from Sidhwan Bet. These diverse responses reveal a scarcity of knowledge, with a thirst for knowledge. This can serve as a point of departure for community education campaigns. The sources of information on cancer that the ASHAs cited were formal training sessions, visits to hospitals, village-level health meetings, and doctor-community health officer (CHO) meetings. Some respondents mentioned meetings and training sessions held at local health centers as common sources of knowledge. Tilhar UP said, "Doctor told us in the training session.” “We are told how to take care of ourselves properly.” Depicting institutional training sessions as the primary source of information.

In the thematic/code family of barriers to screening, ASHAs mentioned fear of infection and sociocultural myths as existing barriers. Some believed "sitting close to a cancer-stricken individual could transmit it to them." They reported that one of the ASHAs commented, "I also had a case 8 years ago, the patient who fell under influence of a baba." The patient did not go to the doctor and stayed at home. Others talked about "stigma and seclusion of the patients," a woman who did not undergo treatment because of religious beliefs and instead consulted a local faith healer. Both myths and misinformation affect help-seeking behaviors, preventing early detection.

Most of the ASHAs reported that they did not screen themselves for oral cancer, breast cancer, or cervical cancer. Although they are community-level primary health educators, some simply replied "No, ma'am," when asked if they ever underwent screening, reflecting a wide gap between what they practiced and what they knew. Few ASHAs had basic knowledge of cancers, because someone in their family or close relatives was affected by a cancer. Another ASHA relative had been operated on for cancer but later succumbed, and another described a colleague who underwent chemotherapy. The personal experience made ASHAs more focused on cancer and its prevention.

ASHAs had poor knowledge about cancer causation and prevention. Some behavioral and lifestyle risk factors, such as tobacco smoking, poor hygiene, and smoking, were known. "… lifestyle habits are the cause of these cancers,” was mentioned by ASHA from Garo Hills. ASHAs also mentioned personal hygiene as a way of preventing cancer. Under the theme Attitudes and Beliefs, the code family “Beliefs About the Prevention” highlighted differing perspectives among ASHA workers regarding the prevention of cancer. Two prominent codes emerged: "Believing and No Belief." Some were confident that cancers can be prevented through awareness and education.

"If we do diagnosis on time, then with treatment, we will get long life," said one ASHA from Pakhowal. Those with "No Belief" reflected a lack of confidence or clarity regarding cancer prevention, especially from the Garo Hills, mentioned, “I heard …, it can't be completely curable.” ASHAs sources of information were a mix of traditional and new media, including posters, pamphlets, workshops, mobile phone advertisements, and health worker education. As one ASHA stated, "Firstly, like on mobiles many advertisements are shown, people who are educated, they try to gather more information through posters."

ASHAs gave several actionable suggestions to improve cancer awareness and screening in their community. The most widely proposed intervention was to organize screening camps, provide free treatments, and community health campaigns through government facilities. Some suggested generating awareness among the community via local religious or cultural festivals. "I prefer that there should be screening camps and free treatments for these cancers in government hospitals." One suggestion was that camps be operated village-wise to enable access and have more people screened.

## Discussion

This qualitative study explored the knowledge, attitudes, and beliefs of ASHAs regarding breast, cervical, and oral cancer in Punjab, Uttar Pradesh, and Meghalaya states of India. Through thematic analysis of FGDs, it was evident that ASHAs have a heterogeneous and incomplete understanding of cancer symptoms, causes, screening behavior, and barriers to care. These results are vital, considering the pivotal role ASHAs occupy in linking rural populations and formal healthcare systems, especially in the context of the increasing incidence of NCDs in India.

Awareness of cancer symptoms

ASHA workers had differing levels of cancer awareness. Whereas some identified major symptoms, like breast lumps, abnormal bleeding per vagina, or oral ulcers, correctly, others had little or no awareness of early warning signs. Awareness of oral cancer was relatively high, particularly of tobacco-related causes, which could indicate the coverage of anti-tobacco campaigns in these areas. This asymmetrical knowledge is in accordance with the findings of Gupta et al., that frontline workers tend to use experiential learning instead of formal cancer education and thus have a fragmented knowledge of symptoms [6]. Misconceptions like smoking can be related to breast cancer, viewing cancer as contagious, etc., were wrong messages, fueling stigma and fear. Dey reported that several urban health centers did not have any visible awareness materials on these cancers during the study, whereas primary health centers (PHCs) were more likely to have them [7]. This disconnect in communication and visibility needs to be filled through uniform awareness initiatives at all care levels.

Sources of cancer information

ASHAs indicated that they received information from a variety of sources, both formal, such as training, community health centres (CHC) meetings, and doctor consultations, and informal, such as mobile advertisements, posters, and peer talk. While this exposure through multiple sources widens the avenues of information, it also carries the risk of misinformation. Similar to the study by Pati et al., stating that ASHA education is influenced by local beliefs or fragmented guidance provided in the process of patient navigation [8]. Our study affirms the necessity for a structured, centralized curriculum for oncology tailored for community health workers (CHWs), especially where specialized care is not readily accessible.

Screening practices and barriers

Despite the recognition of the need for early detection, none of the ASHAs had ever screened themselves for common cancers. The commonly reported obstacles were fear of infection, insufficient time, cultural stigma, and fear of being shunned by society (ostracization). Fatalistic attitudes and reliance on traditional healers are identified as major barriers to receiving adequate and timely medical care.

Further, Winnie et al. (2019) proposed a Hong Kong theory framework where CHWs can enhance awareness of breast and cervical cancers, but only if pre-requisites of access/proximity of services and appropriate training were met; without them, the motivation to engage with the community is significantly diminished [9]. In India, Elias et al. reported critical gaps in the delivery of common NCDs like diabetes and hypertension care in South India. These gaps were attributed to a lack of continuum of care, and for cancer care, the bottlenecks would be even greater [10]. A time-motion study conducted in Assam by Oswal et al. reported that ASHAs and auxiliary nurse midwives (ANMs) spent only 11% and 4%, respectively, of their working hours on NCD-related activities, which would be even lower prioritization for cancer prevention [11]. This knowledge-practice gap is similar to that reported by Palo et al., where even literate patients are reluctant to seek cancer care due to fear of judgment, misinformation, stigma, or system constraints [12].

Impact of personal experience on awareness and involvement

ASHAs with direct personal or family experience with cancer exhibit higher empathy, cancer awareness, and greater motivation to disseminate information to their own communities. They tend to tell stories of suffering, survival, or loss, bringing in an emotional involvement in cancer education. Nair et al. showed that participatory models, e.g., participatory learning and action (PLA), improve health outcomes and may similarly be modified for cancer education [13]. Involving ASHAs through such participatory platforms enhances their role in cancer advocacy.

Attitudes toward cancer prevention and early detection

There were varied beliefs regarding cancer prevention. While most ASHAs felt confident about prevention by way of better hygiene, healthy living, and early screening, some had more fatalistic or pessimistic attitudes. However, there existed a strong consensus regarding the advantage of early detection - most respondents concurred that early diagnosis increases survival and decreases treatment burden. This means that although baseline knowledge is present, it needs to be complemented with correct, scientific information to counter existing myths. Equipping ASHAs with knowledge as well as self-efficacy helps them become proactive facilitators for community involvement in cancer screening programs.

Trusted sources and educational tools

ASHAs identified several sources of reliable health information: physicians, CHOs, posters, pamphlets, workshops, and even mobile phone adverts. They indicate the importance of a multi-strategic cancer education program that blends interpersonal communication and mass media approaches.

The trend of social media influencers with untested, sensationalized, or poorly designed messages creates confusion. There is a need for standardized and culturally appropriate IEC materials.

Community-based recommendations and policy implications

The grassroots-level suggestions for increasing awareness and screening included door-to-door counseling, conducting free screening camps, involvement of faith-based institutions, and encouraging greater healthcare access. Secondly, the Ayushman Bharat’s Health and Wellness Centre (HWC) can enhance access to integrated primary care, including cancer screening services, providing a continuum of care, including tertiary care. Ved et al. noted that HWCs offer a platform where ASHAs can be empowered to function better as primary care providers for linking communities with key NCD services [14]. According to the National Health Mission (2013), integration of screening, early diagnosis, and management as close to the community as possible will more than likely reduce the need for higher-level care and lower out-of-pocket healthcare costs [15]. As Arora et al. highlighted, developing strong linkages between the community and the health system and incorporating education for NCDs in national health missions are indispensable [16]. Equipping ASHAs with adequate training, supportive monitoring, and development of culturally appropriate materials is essential to the effectiveness of any cancer prevention program at the grassroots level.

Limitations

Although this study provides valuable insights into ASHAs’ perceptions of cancer awareness and screening, several limitations should be acknowledged. The findings are based on qualitative data collected from selected districts in Punjab, Uttar Pradesh, and Meghalaya, which may limit the transferability of the results to other regions with different health system capacities and socio-cultural contexts. In addition, the distribution of participants across the study sites was uneven, with a larger number of ASHAs recruited from Ludhiana district in Punjab compared to participants from Meghalaya and Uttar Pradesh. This variation was primarily due to the availability of active ASHAs and the ease of coordinating FGDs during the data collection period. Consequently, perspectives from certain regions may be underrepresented. Furthermore, the purposive sampling of active ASHAs may have excluded those who are less engaged in health programs, potentially leading to an underestimation of existing knowledge gaps. The group dynamics inherent to FGDs may also have influenced responses, as more vocal participants may have shaped the discussion while others may have been less inclined to express their views. Future research should include larger and more geographically diverse samples across multiple states to improve the breadth of perspectives. Incorporating methodological triangulation, such as combining qualitative findings with surveys, direct observation, and service utilization data, may provide a more comprehensive understanding of ASHAs’ roles in cancer prevention and screening. Evaluating the effectiveness of structured cancer-specific training modules, refresher programs, and supportive supervision mechanisms would help identify sustainable capacity-building strategies. Further studies should also explore the feasibility of integrating cancer screening promotion within existing NCD platforms, particularly through HWCs, and assess the role of digital decision-support tools and culturally tailored information, education, and communication (IEC) materials in improving ASHAs’ confidence and community screening uptake.

## Conclusions

The findings of this study indicate that although ASHA workers possess some awareness of cancer symptoms and prevention, their knowledge remains fragmented and influenced by personal beliefs, local misconceptions, and variations in training. The FGDs revealed that stigma, fear, and practical barriers continue to limit the promotion and uptake of cancer screening within communities. These findings highlight the need for structured and standardized training programs on cancer awareness, screening, and referral pathways for ASHA workers. Strengthening access to reliable information resources and institutional support may enhance their capacity to effectively communicate cancer-related information and encourage early detection practices. Providing ASHAs with evidence-based and culturally appropriate educational tools could help bridge the knowledge-practice gap and improve community-level cancer prevention and screening outcomes.

## Appendices

Semi-structured interview guide

A. General Awareness

1. What do you know about breast cancer, cervical cancer, and oral cancer?

2. How would you describe the current level of awareness and knowledge about these cancers among women in your community?

B. Screening and Prevention

1. What do you think are the main barriers that prevent women from undergoing screening for these cancers?

2. What do you know about screening tests for cervical cancer, such as the Pap smear and HPV testing?

3. In your opinion, what measures can help in preventing these cancers at the community level?

C. Personal Experience

1. Have you ever encountered or supported someone diagnosed with breast, cervical or oral cancer?

2. If yes, could you share your experience and how it affected you and the person diagnosed?

D. Attitudes and Beliefs

1. Do you believe that breast, cervical and oral cancers can be prevented or detected early?

2. What are your perceptions regarding the effectiveness of early detection and screening programmes?

E. Information and Education

1. What type of training or educational programmes do you feel ASHAs need to improve cancer awareness and screening uptake in the community?

2. What educational methods (talks, visual aids, community meetings) do you think would be most effective?

F. Concluding Question

1. Do you have any additional comments or suggestions regarding awareness, prevention, or screening of breast, cervical, and oral cancers?

One-Line Methodological Description

A customised semi-structured interview guide was developed, pilot tested, and validated to explore cancer awareness, perceptions, and screening-related experiences among ASHAs.
